# Pregnancy environment, and not preconception, leads to fetal growth restriction and congenital abnormalities associated with diabetes

**DOI:** 10.1038/s41598-020-69247-w

**Published:** 2020-07-23

**Authors:** Pai-Jong Stacy Tsai, Yasuhiro Yamauchi, Jonathan M. Riel, Monika A. Ward

**Affiliations:** 1 Institute for Biogenesis Research, John A. Burns School of Medicine, University of Hawaii, 1960 East-West Rd, Honolulu, HI 96822 USA; 2 Department of Obstetrics, Gynecology, and Women’s Health, John A. Burns School of Medicine, University of Hawaii, Kapi’olani Medical Center for Women & Children, 1319 Punahou Street, Honolulu, HI 96826 USA

**Keywords:** Developmental biology, Medical research

## Abstract

Maternal diabetes can lead to pregnancy complications and impaired fetal development. The goal of this study was to use a mouse model of reciprocal embryo transfer to distinguish between the preconception and gestational effects of diabetes. To induce diabetes female mice were injected with a single high dose of streptozotocin and 3 weeks thereafter used as oocyte donors for in vitro fertilization (IVF) and as recipients for embryo transfer. Following IVF embryos were cultured to the blastocyst stage in vitro or transferred to diabetic and non-diabetic recipients. Diabetic and non-diabetic females did not differ in regard to the number of oocytes obtained after ovarian stimulation, oocytes ability to become fertilized, and embryo development in vitro. However, diabetic females displayed impaired responsiveness to superovulation. Reciprocal embryo transfer resulted in similar incidence of live fetuses and abortions, and no changes in placental size. However, fetuses carried by diabetic recipients were smaller compared to those carried by non-diabetic recipients, regardless hyperglycemia status of oocyte donors. Congenital abnormalities were observed only among the fetuses carried by diabetic recipients. The findings support that the diabetic status during pregnancy, and not the preconception effect of diabetes on oogenesis, leads to fetal growth restriction and congenital deformities.

## Introduction

The incidence of diabetes is on the rise world-wide affecting women of child-bearing age^1–3^. Complications arising from the effects of maternal diabetes on fetal development include growth delay, miscarriage, stillbirth, macrosomia, and congenital malformations^4–7^. Higher risk for preterm delivery was also reported^8–10^. Finally, preexisting diabetes lead to increased incidence of maternal complications such as hypoglycemia, gestational hypertension, preeclampsia, preterm labor and Caesarean delivery, as well as other comorbidities^1,11–13^. Early glycemic control in pregnancy is crucial for decreasing adverse outcomes.

Rodent models of pregestational diabetes are usually developed by using diabetogenic agents, such as streptozotocin (STZ), to induce diabetes in females. Diabetes induced by a single high dose STZ injection has been previously shown to produce severe hyperglycemia similar to a type 1 diabetes^14^. Mouse models of diabetes have already significantly contributed to the understanding of etiology and progression of diabetes^15^. These models permit the control of the genetic and environmental factors that may influence the development, establishment, and complications of disease. There is an abundance of evidence indicating the presence of multiple female reproductive problems in murine models of diabetes, including delayed oocyte maturation, disruption of metabolic processes and of the epigenetic code in oocytes, reduced ovulation rates, impaired implantation, poor embryo development, and fetal malformations^16–22^. These reports support the model that preconception diabetes has effects on oogenesis, female fertility, and pregnancy outcome.

In humans, the term gestational diabetes refers to diabetes that manifest for the first-time during pregnancy, without preceding hyperglycemia that might have influenced oogenesis. Thus, any observed negative effects reflect the gestational effects of hyperglycemia. However, when diabetic women become pregnant, their diabetic status can potentially affect both the preconception and the pregnancy.

Animal models of diabetes offer a unique opportunity to distinguish between the effects on oogenesis (pre-conception) and on embryonic/fetal development in utero (gestation). Specifically, using the reciprocal embryo transfer mouse model of diabetes, in which embryos from diabetic females are transferred to non-diabetic recipient, and vice-versa, it is possible to test for the effects of preconception and gestation, independently from each other.

Four prior studies utilizing this approach in mice and in the context of diabetes or high fat diet (HFD) provided interesting yet variable results.

In the first study, when embryos, either zygotes or blastocysts, from STZ-induced diabetic females were transferred to non-diabetic recipients, the resulting fetuses exhibited retarded growth and higher incidence of malformations, suggesting of a preconception rather than a gestational effect^23^. This report also provided evidence that time of exposure to metabolic changes resulting from diabetes is critical and that a window between conception and implantation is highly sensitive.

In the second study, high incidence of fetal anomalies observed after natural mating of nonobese diabetic (NOD) mice decreased from 40 to 14% when the zygotes were flushed, cultured to morula/early blastocyst in vitro, and then transferred to uteri of wild-type control recipients. At the same time, when embryos from wild-type mice were transferred to uteri of diabetic NOD recipients, incidence of fetal abnormalities increased from 1 to 40%, suggesting that diabetic gestational environment has deleterious effects on fetal development ^24^.

In the third study, when reciprocal 2-cell embryo transfer was applied after pregestational exposure of females to HFD, both the transfer of embryos from HFD-fed females to the uteri of control females and the transfer of embryos from control females to uteri of HFD-fed females led to impaired placental and fetal growth, suggesting contribution of both preconception and gestation^25^.

Finally, in the fourth study, when embryos from non-diabetic females were transferred to diabetic recipients, but not when embryos from diabetic females were transferred to non-diabetic recipients, methylation patterns and expression of imprinted genes in placentas and whole fetuses were altered, implying a gestational effect^26^.

Together, these studies support the model that both preconception and gestation effects of diabetes may contribute to alteration of pregnancy outcome. The effects of STZ-induced diabetes on gestation, independent from preconception effect, have not yet been investigated. Also, in all prior studies conception was achieved in vivo and thus by necessity extending the preconception effect through fertilization and until embryos were flushed. Thus, it remains unknown whether preconception effect on oogenesis only bears consequences for embryonic/fetal development.

To address these gaps in knowledge we induced diabetes in CD1 mice by a single, high dose STZ injection and examined preconception and gestational effects of hyperglycemia on perinatal outcomes using in vitro fertilization that allows for a conception outside the direct influence of diabetes, followed by 2-cell reciprocal embryo transfer. Our data revealed that pregnancy environment, and not preconception, is causative of adverse reproductive outcomes associated with diabetes.

## Results

### Single injection of STZ results in severe diabetes

To induce DM mice were subjected to a single SZT injection. To assess the effect of STZ injection effectiveness in inducement of DM three cohorts of mice (Cohort 1–3) consisted of two groups, STZ-injected (STZ) and vehicle-injected (CON). The mice were monitored for 21 days post injection. In all three cohorts there were no differences between two examined groups in body weight changes. Body weight dropped slightly after injection (Fig. S1A) and then increased steadily (Fig. S1A,C,E). Vehicle-injected mice displayed stable blood glucose levels averaging 130 mg/dL, 152 mg/dL and 160 mg/dL, for Cohort 1, 2 and 3, respectively (Fig. S1B,D,F). The STZ injection increased blood glucose, with significantly higher levels compared to controls starting as early as 6 (Fig. S1F) or 14–15 days (Fig. S1B, D) post-injection, and on day 21 averaging 443 mg/dL, 308 mg/dL, and 401 mg/dL, for Cohort 1, 2 and 3, respectively (Fig. S1B,D,F). Four additional cohorts (Cohort 4–7) included only SZT-injected mice. Although response to STZ injection was variable among mice, females with blood glucose levels well above 250 mg/dL were consistently obtained on day 21 post-injection and the glucose levels continued to increase or remained stably high with passing time up, occasionally reaching as high as > 600 mg/dL (Fig. S2). Mice with blood glucose levels > 250 mg/dL were used as oocyte donors for IVF or as surrogate mothers for embryo transfer.

### Oocyte number, fertilization efficiency, and preimplantation development are not affected by diabetes

**Figure 1:**
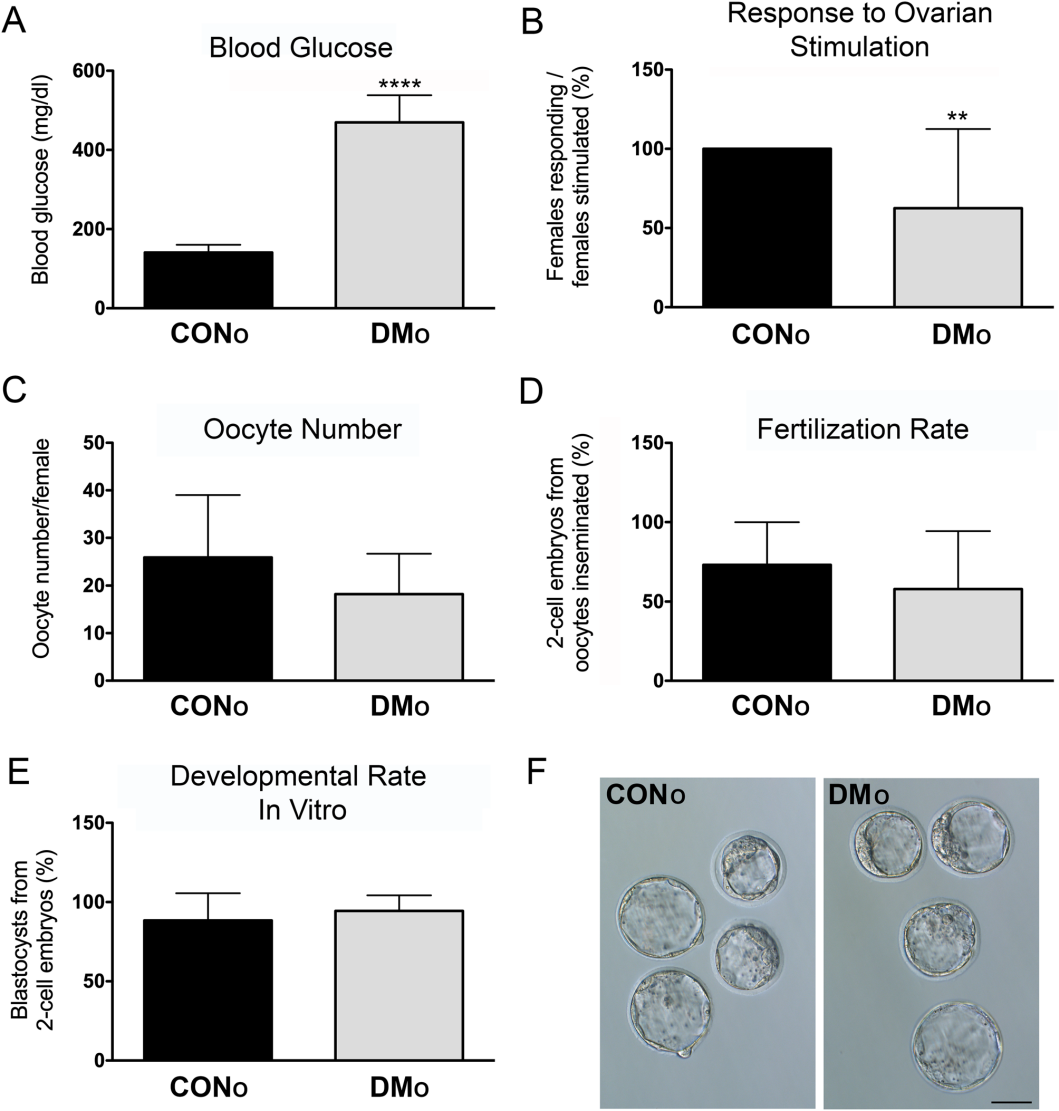
Effects of DM on in vitro fertilization and in vitro embryo development. Diabetic (DM_O_) and non-diabetic (CON_O_) females provided oocytes for in vitro fertilization. DM_O_ females had higher blood glucose level (**A**) and decreased response to hormonal ovarian stimulation (**B**) when compared to CON_O_ females. However, the average oocyte number per female (**C**), fertilization rate (**D**) and in developmental rate in vitro (**E**) were similar in both groups. In vitro produced embryos developed to healthy, good looking expanded blastocysts (F). Graphs are average ± SDev with n = 16 and n = 15 (**A**, **B**) and n = 10 and n = 15 (**C**–**E**) for DM_O_ and CON_O_, respectively. Statistical significance (t-test): **P < 0.01; ****P < 0.0001. For statistical analyses all percentages were transformed to angles. Scale bar in (**F**), 50 µm. For the data in (**B**) females were considered individually: female that responded, 1/1, 100% and female that did not respond, 0/1, 0%. Raw data are shown in Table S1.

Two groups of females, diabetic (DM_O_) and non-diabetic (CON_O_) were used as oocyte donors for in vitro fertilization, performed in three experimental replicates (Table S1). Females from the DM_O_ group had significantly higher average blood glucose level (460 mg/dL) as compared to CON_O_ (141 mg/dL) (Fig. 1A, P < 0.0001). Females from the DM_O_ group did not always respond to induced ovarian stimulation, with only 10 out of 16 being responsive, as compared to 15 out of 15 CON_O_ females yielding oocytes (Fig. 1B, P = 0.007). The average oocyte number per female for the females that did respond was lower for the DM_O_ group than for the CON_O_ group, 18 vs. 26, but the difference was not statistically significant (Fig. 1C, P = 0.1174). When the oocytes from DM_O_ and CON_O_ females underwent in vitro fertilization, 57% and 75% developed to 2-cell embryos, respectively. Although the proportion of the oocytes that developed to the 2-cell stage was lower for the DM_O_ than the CON_O_ females, the difference was not statistically significant (Fig. 1D, P = 0.3174) likely due to the differences between individual females (Table S1). Embryo development in vitro was also unaffected by the diabetic status of the oocyte donors, with similar proportions of 2-cell embryos developing to the blastocyst stage in vitro (94% and 88% for DM_O_ and CON_O_, respectively (Fig. 1E, P = 0.1419) and yielding high quality expanded blastocysts (Fig. 1F).

### In vivo development under diabetic environment results in decreased fetal body weight and size, and increased incidence of congenital abnormalities

**Figure 2:**
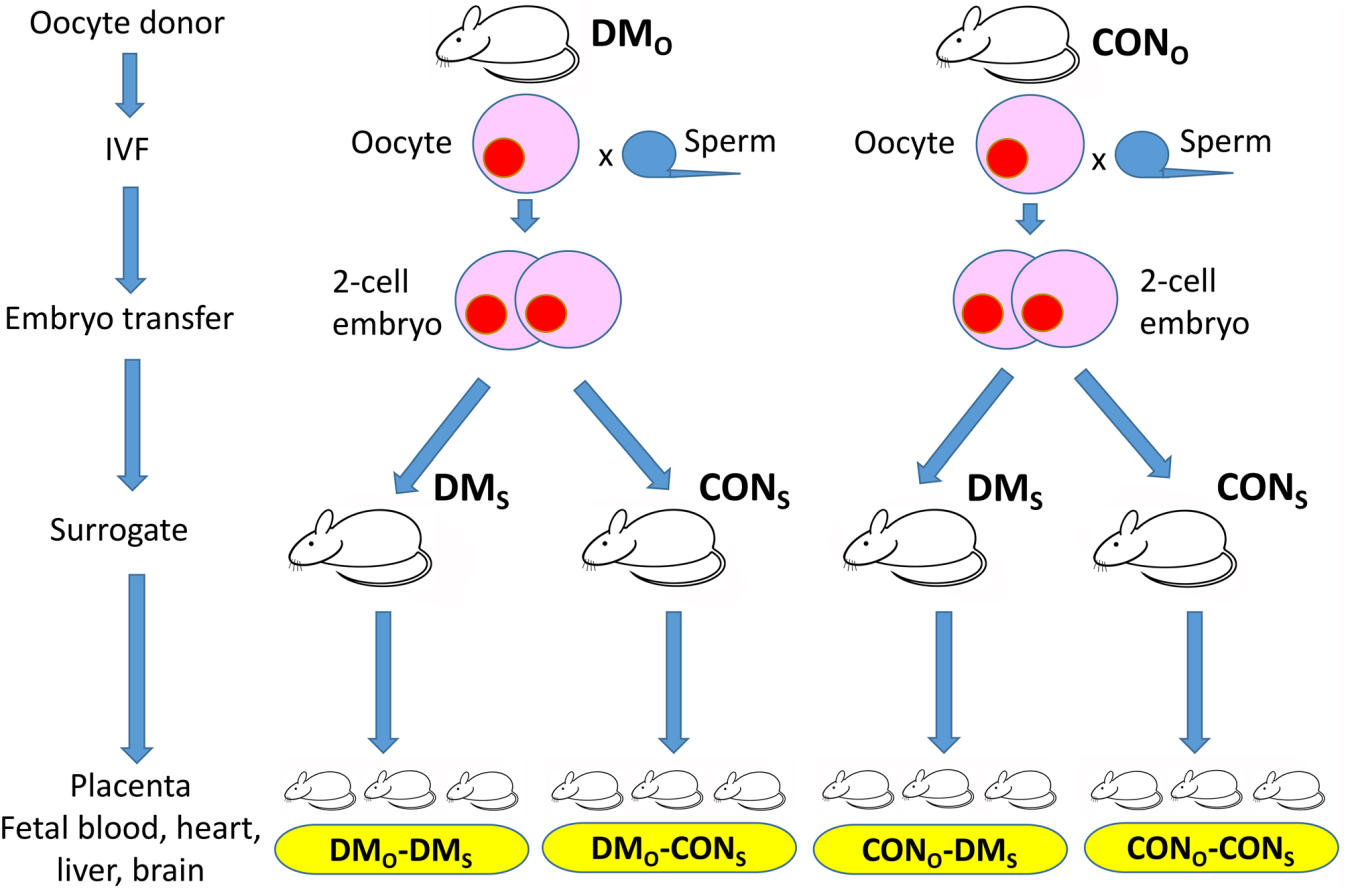
Reciprocal embryo transfer. Embryos obtained after in vitro fertilization with oocytes from diabetic (DM_O_) or non-diabetic (CON_O)_ females were transferred into the oviducts of diabetic (DM_S_) or non-diabetic (CON_S_) surrogates. At 18.5 days of pregnancy caesarian section was performed and live fetuses and abortions were obtained and scored. The four experimental groups (CON_O_-CON_S_, DM_O_-CON_S_, CON_O_-DM_S_, and DM_O_-DM_S_) were compared in regard to fetal developmental potential and provided fetal and placental tissues for banking.

**Figure 3:**
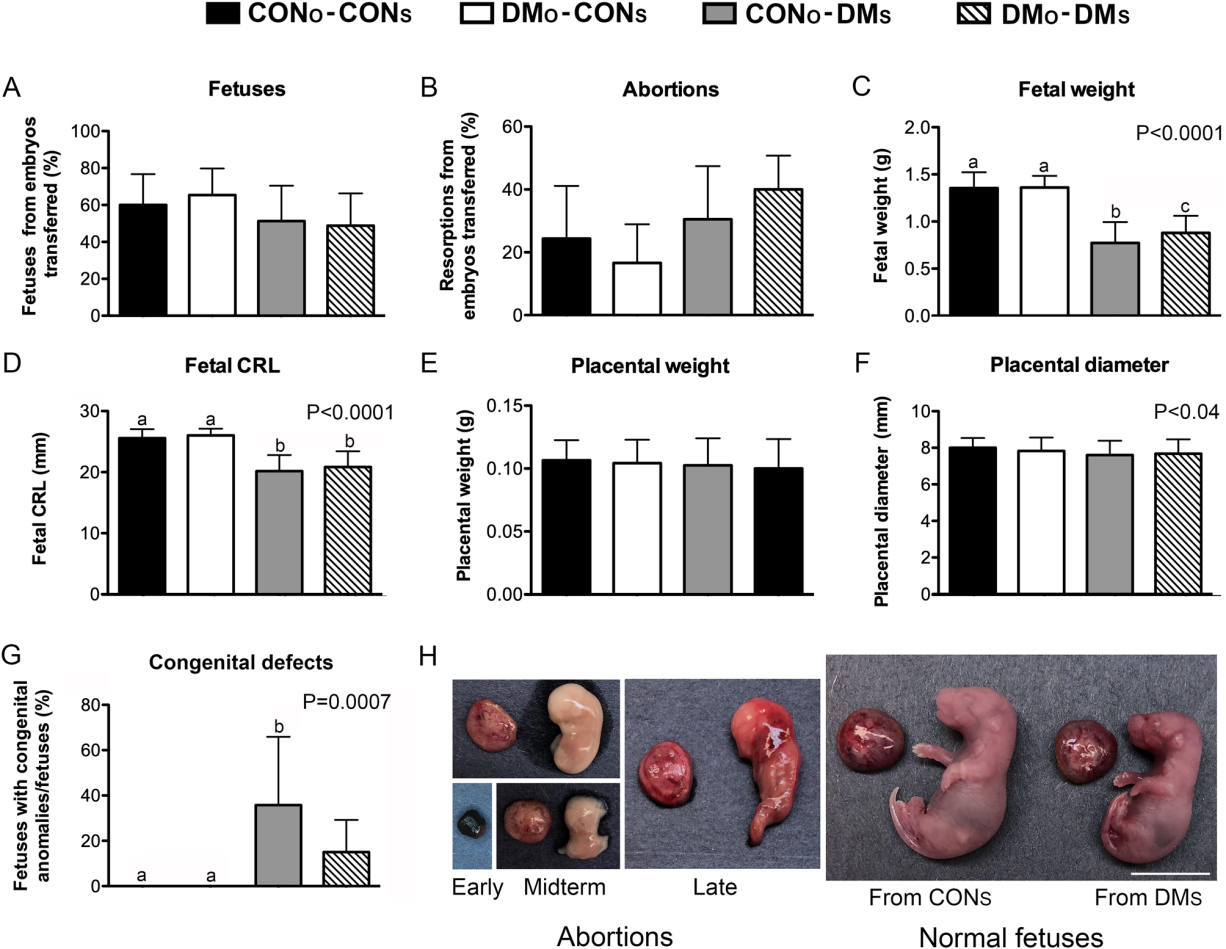
Effects of DM on post-implantation development. Embryos obtained after in vitro fertilization with oocytes from diabetic (DM_O_) or non-diabetic (CON_O)_ females were transferred into the oviducts of diabetic (DM_S_) or non-diabetic (CON_S_) surrogates. At 18.5 days of pregnancy caesarian section was performed and number of live fetuses and resorption sites were scored. Fetuses and placentas were weighted and measured. Four experimental groups (CON_O_-CON_S_, DM_O_-CON_S_, CON_O_-DM_S_, and DM_O_-DM_S_) were compared in regard to proportion of fetuses (**A**) and resorption sites (**B**) from embryos transferred, fetal weight (**C**), fetal crown to rump length (CRL, **D**), placental weight (**E**), placental diameter (**F**), and incidences of fetuses with congenital defects (**G**). The data were analyzed with 1-way ANOVA analysis of variance with post-hoc Bonferroni test for multiple paired comparison. There were no differences between the groups in incidence of fetuses and abortions in ANOVA (P = 0.408 and P = 0.139, respectively) and post-hoc test. When the data shown in (**A**) and (**B**) were analyzed by t-test paired comparison, more sensitive than ANOVA, only one significant difference was noted (Abortions, DM_O_-DM_S_ vs. DM_O_-CON_S_, P = 0.027). Fetal weight and CRL were significantly decreased with DM_O_ surrogates as shown by ANOVA (P < 0.0001) and post-hoc test (**A** vs. **B** and **C**, P < 0.0001; **B** vs. **B**, P < 0.05). Placental diameter was also slightly decreased with DM_O_ surrogates in ANOVA (P < 0.04), with no differences between groups in paired comparison. Congenital defects were only observed among fetuses from diabetic surrogates (ANOVA P = 0.0007, post-hoc **A** vs. **B**, P < 0.01). When the data shown in (**G**) were also analyzed by t-test paired comparison the difference was noted also between DM_O_-DM_S_ vs. CON_O_-CON_S_ and DM_O_-CON_S_ (P = 0.012) and the difference between CON_O_-DM_S_ vs. CON_O_-CON_S_ and DM_O_-CON_S_ increased (P = 0.005). In (**H**), the examples of early, midterm and late abortions and normal fetuses and placentas from DM_S_ are shown. Scale in H, 1 cm (except for bottom most left panel which is not to scale). Graphs are average ± SDev with n = 5, 5, 4, and 5 (surrogates) in (**A**) and (**B**), and n = 54, 47, 37, and 44 (fetuses or placentas) in (**C**) to (**G**), for CON_O_-CON_S_, DM_O_-CON_S_, CON_O_-DM_S_, and DM_O_-DM_S_, respectively. For statistical analyses percentages were transformed to angles. Raw data for (**A**), (**B**) and (**G**) are shown in Table S3.

Embryos obtained after in vitro fertilization were cryopreserved for subsequent embryo transfer. Cryopreservation was necessary because of difficulties with having appropriate diabetic and non-diabetic surrogates ready at the time required for fresh embryo transfer. The cryopreservation of IVF-derived 2-cell embryos does not influence reproductive outcome (Table S2). Embryos obtained after in vitro fertilization with oocytes from diabetic (DM_O_) or non-diabetic (CON_O_) females were transferred into the oviducts of diabetic (DM_S_) or non-diabetic (CON_S_) surrogates. Four experimental groups (CON_O_-CON_S_, DM_O_-CON_S_, CON_O_-DM_S_, and DM_O_-DM_S_) were compared (Figs. 2, 3, Table S3). On day 18.5 of pregnancy caesarean section was performed and numbers of live fetuses and resorption sites were scored. Fetuses and placentas were weighted, measured, and assessed for normalcy.

There were no differences between groups in regard to the proportions of live fetuses and abortions (Fig. 3A,B, Table S3). Between 49 and 66% of transferred embryos developed to viable fetuses, and between 17 and 40% of transferred embryos aborted. A trend towards a lower incidence of fetuses and a higher incidence of abortions was noted with diabetic surrogates but the differences were not statistically significant (Fig. 3A,B, P = 0.408 and P = 0.139, respectively). Among abortions, both early, midterm and late abortions were observed, and abnormalities were frequently noted (Fig. 3H).

Fetuses carried by diabetic surrogates displayed significantly decreased body weight and length when compared to fetuses carried by non-diabetic surrogates (Fig. 3C,D). This was observed regardless of the diabetic status of oocyte donors. Placental development was overall similar among groups, with a small, but significant differences in placental diameter (Fig. 3E,F). Sex distribution (males vs. females) among fetuses was similar across the groups (Fig. S3).

**Figure 4:**
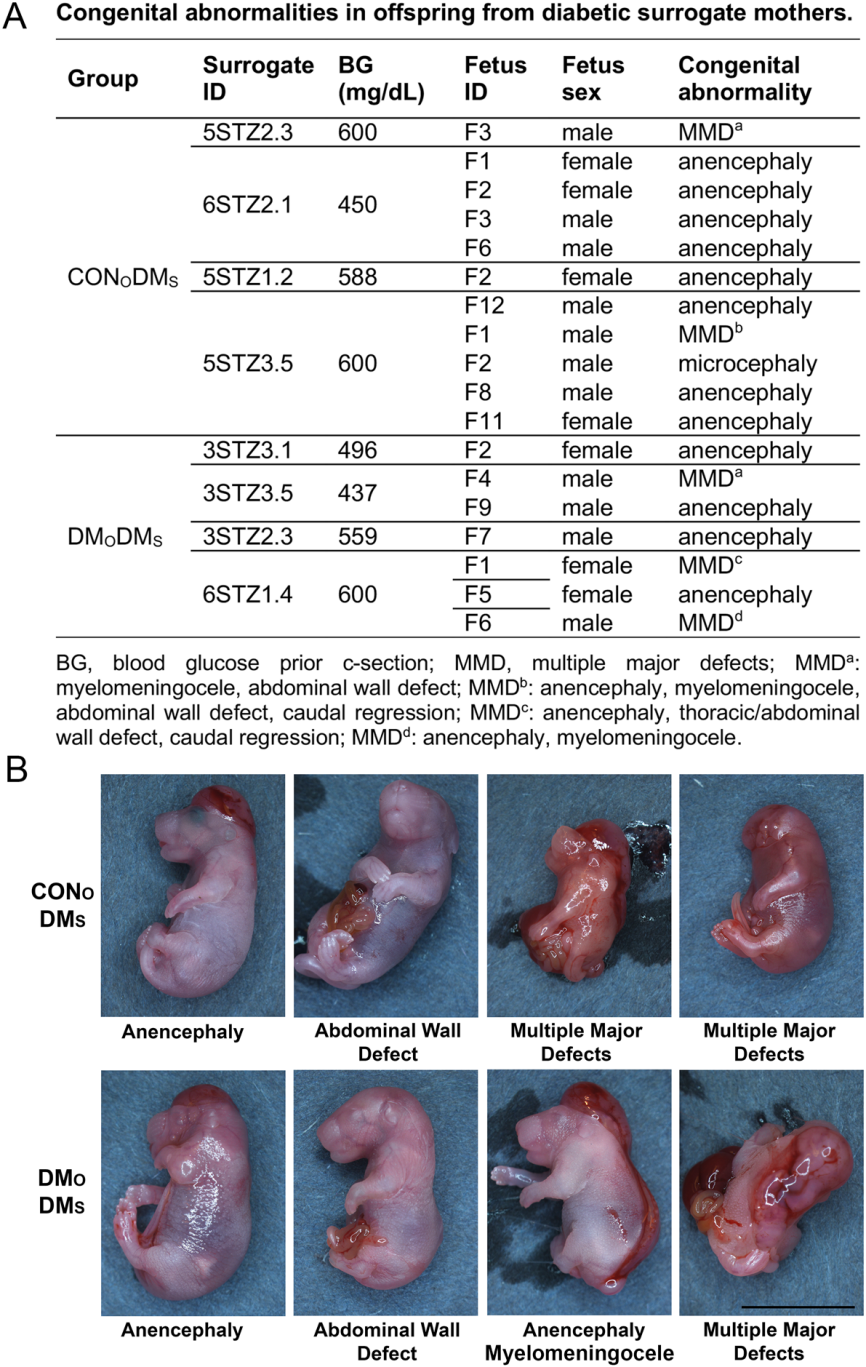
Congenital malformations of fetuses from diabetic females. Fetuses derived from embryos produced with non-diabetic (CON_O_) and diabetic (DM_O_) oocyte donors transferred to diabetic surrogate mothers (DM_O_) displayed various congenital malformations. Distribution of congenital abnormality types is shown in (**A**). The examples of fetuses with malformations are shown in (**B**). Scale, 1 cm.

The viable fetuses were scored for normalcy. All fetuses that developed in uteri of non-diabetic recipients were normal. However, among fetuses that developed in uteri of diabetic surrogates several displayed congenital malformations: 11 out of 37 (30%) for CON_O_-DM_S_ group and 7 out of 44 (16%) for DM_O_-DM_S_ group (Fig. 3G, Table S3). Although DM_O_-DM_S_ group yielded fewer abnormal fetuses than CON_O_-DM_S_ group, the defects were more severe with 43% (3/7) fetuses displaying multiple major defects as compared to 18% (2/11). The most frequently observed defect was anencephaly, with 83% (15/18) abnormal fetuses displaying this anomaly (Fig. 4). Other abnormalities included isolated microcephaly (1/18) and defects that appeared in combination and included myelomeningocele (3/18), abdominal wall defects (4/18), microcephaly (1/18), caudal regression (2/18), and thoracic wall defect (1/18). Although deformities were sometimes severe, fetuses were alive, evidenced by a persisting heartbeat (Movie S1).

## Discussion

The goal of this study was to use a mouse model of reciprocal embryo transfer to distinguish between the preconception and gestational effects of diabetes. The data support that the diabetic status during pregnancy, and not the preconception effect of diabetes on oogenesis, leads to fetal growth restriction and congenital deformities.

In the first part of the study we examined the effects of STZ-induced preconception diabetes on oogenesis, ovulation, fertilization, and preimplantation embryo development. We have shown that the oocyte number, the ability of oocytes to become fertilized in vitro, and preimplantation embryo development in vitro were unaffected. This stands in contrast to previous studies reporting that superovulated STZ-treated females yielded fewer oocytes when compared with controls, and that these oocytes were developmentally delayed or displayed various anomalies including meiotic spindle defects, chromosome misalignment and aneuploidy, and increased granulosa/cumulus cells apoptosis, and that these defect could be ameliorated by insulin treatment or islet transplantation^17,18,27^. In these studies, the ovulation induction was done on the fourth day after the STZ injection while in our work females were stimulated a minimum of 3 weeks after the injection. Streptozotocin exerts cytotoxic effect on pancreatic β cells^28^ and is eliminated within 48 h of ingestion^29^ so its acute toxicity is short-lived. The hyperglycemic state that becomes established by acute STZ toxicity drives further deterioration of pancreatic β cells^30^ and affects other organs, like liver, kidney, brain, heart and muscles^31–33^. The oogenesis/oocyte defects described in these three studies therefore represent a response to short duration of STZ-induced diabetic hyperglycemia. However, Lee et al.^34^ used a similar experimental regime as we did in our study, with ovarian stimulation taking place 3 weeks after STZ injection, and observed impairment in folliculogenesis, oogenesis, and preimplantation embryogenesis. Moreover, Akita mice which harbor a mutation resulting in pancreatic beta cell dysfunction that renders them chronically diabetic, were shown to display a similar phenotype as the STZ-injected mice^17,34^. Thus, both short and long exposure to hyperglycemia can lead to defects in oocyte maturation and ovulation. The duration of exposure does bear an effect as different outcomes were noted with insulin rescue initiated at different times post STZ; an effective rescue was achieved when insulin was delivered 2 days after STZ treatment^18^ while lower effectiveness was noted with delivery on day 6 days after STZ^27^.

Existing literature suggests that preimplantation embryo development is negatively influenced by the diabetic status of the mother. In past studies the diabetes was chemically induced in female mice prior to mating and the flushed embryos were examined for the developmental progression. Embryo development was delayed, and decreased embryonic viability and growth, and impaired genetic integrity were also noted^16,35–37^. We did not observe deficiencies in preimplantation embryonic development, with the 2-cell embryos derived from oocytes form diabetic and non-diabetic females developing to the blastocyst stage with similar efficiency. However, in our study both the fertilization and the entire duration of embryonic development to the blastocyst stage took place in vitro under standard culture conditions. Thus, the diabetic state of the mother directly influenced oocyte development, growth, and ovulation, but not the periconception period, which was previously indicated as particularly sensitive^16,38^.

The only effect of STZ-induced preconception diabetes that we observed was impaired female responsiveness to superovulation. Only 10 out of 16 diabetic females responded to superovulation, while non-diabetic females were all (15/15) responsive. In the majority of past studies, in which a superovulation regime was applied to STZ-diabetic mice information on female responsiveness was omitted^39,40^. We identified only one report providing this information, with no decrease noted in STZ-injected C57BL/6 females compared to controls^34^. These authors noted that follicle growth was properly stimulated by exogenous gonadotropins. However, the histological assessment revealed that ovaries of STZ-injected mice contained “trapped” oocytes, suggesting impairment in ovulation process. In our study, at the time of euthanasia, the ovaries from diabetic females did not display any visible defects and were similar in gross assessment to the ovaries of control females. It is possible that more detailed investigation would reveal defects in oogenesis/folliculogenesis progression and/or “trapped” oocytes. Future studies will address these possibilities. It will also be worth checking whether oocytes from the non-responsive diabetic females could be matured in vitro and then used for IVF yielding blastocysts and fetuses after embryo transfer.

In the second part of the study we used the reciprocal 2-cell embryo transfer to examine the effects of hyperglycemia on post-implantation embryonic/fetal development. Two-cell embryos generated in vitro with oocytes from diabetic and non-diabetic females were transferred to the oviducts of the diabetic and non-diabetic recipients. This allowed us to independently examine four experimental groups, with exposure to a diabetic environment (1) during preconception only; (2) during gestation only; (3) during both preconception and gestation; (4) and with no exposure.

We did not observe the differences between these four groups in regard to the incidence of embryos that implanted, aborted, or developed to viable near-term (18.5 days post-coitum, dpc) fetuses. However, fetuses developing in the uteri of diabetic recipients, regardless the diabetic status of the oocyte donors, were smaller when compared to fetuses developing under the non-diabetic maternal environment. Fetal growth reduction in diabetic pregnancies have been reported before by many groups and for multiple rodent species, including mice^16,22,23^ and rats^41–43^. The difference between our study and the previous reports in regard to implantation and fetal development to term may, once again, reflect the differences in exposure to diabetic environment. In our study, the fertilization and the 24 h post conception took place in vitro, under neutral, non-diabetic culture conditions. Perhaps exposure to hyperglycemia during this sensitive window is necessary to impair implantation leading to embryonic/fetal loss. In support of this notion, it was reported before that when flushed embryos from diabetic females were transferred to non-diabetic recipients, the resulting fetuses had higher incidence of malformations compared to controls represented by embryos from non-diabetic females transferred to non-diabetic recipients^23^. The zygote transfer did not lead to an increase in abortions or a decrease in implantations but the transfer at the blastocyst stage did^23^. Also, control 2-cell embryos that were cultured in vitro in higher glucose levels, mimicking one aspect of the diabetic condition, prior to transfer resulted in fewer implantations, a higher incidence of abortions, and decreased fetal growth, but no fetal deformities^23^. Although in this past study the preconception diabetes might have contributed to observed phenotypes, it is clear that the time just after fertilization and up to the blastocyst stage is important and may allow for both enhancing and attenuating the effects of prior or existing hyperglycemia.

The fact that we did not observe embryonic/fetal developmental problems after preconception exposure to diabetes and with fertilization achieved in vitro points to the unusual possibility that assisted reproduction technologies, i.e. IVF followed by in vitro embryo culture, may rescue some of the negative effects of diabetes. Clearly, more work is necessary to test this idea, including detailed investigations of fetal developmental potential after in vivo and in vitro conceptions combined with varying times of in vitro culture prior to transfer. If ART can indeed ameliorate some of the negative effects of diabetes, this could lead to new strategies for managements of fertility of diabetic women.

The fetuses developing in uteri of diabetic females were not only smaller than those developing in uteri of healthy females, but some of them displayed congenital abnormalities. We designed the study to be able to quantify implantation and live fetus rates and did not attempt to foster c-section derived fetuses. If we did, we expect that the deformed fetuses would not survive either due to the severity of defects (see Fig. 4) or because their foster moms would cannibalize them as usually happens in case of abnormal pups. The low birth fetuses would likely survive to adulthood if properly taken care about by their foster mothers. In our past work with various transgenic/mutant mice we often dealt with low birth weight pups, including those delivered via c-section and fostered, and have seen them develop well along normal weight pups.

When we assessed the fetuses from four groups resulting from the reciprocal embryo transfer for their normalcy, malformations were noted only when the fetuses were developing in the uteri of diabetic recipients, regardless the diabetic status of oocyte donors. The most common malformation was anencephaly, with few multiple major defects including a combination of myelomeningocele, caudal regression, and abdominal wall defect. In humans, multiple organ systems are susceptible to teratogenic effects of diabetes. Studies of congenital anomalies in infants of diabetic mothers have shown cardiovascular, genitourinary, musculoskeletal, and other malformations. Available data suggest that the most affected are the cardiovascular and central nervous systems (reviewed by^6,44–46^). Although caudal regression was reported having the strongest association with diabetes, it is quite rare. Congenital heart disease represents a more serious problem because its overall incidence is higher^46–48^.

Our analyses of fetal normalcy were based on a gross morphological inspection alone and did not allow to identify and characterize internal defects, such as specific cardiovascular abnormalities, brain alterations, or cellular abnormalities in overtly normal placentas. However, for all fetuses derived from the reciprocal embryo transfer experiments we collected tissue samples (placenta and fetal heart, liver, brain and blood) for future analyses. These banked tissues should also enable investigations of the molecular changes associated with diabetes that may advance understanding of the mechanisms underlying the effects of hyperglycemia on pregnancy outcome.

To summarize, we demonstrated that in the mouse the diabetic status of the mother during pregnancy, and not the preconception effect of diabetes on oogenesis, leads to fetal growth restriction and congenital deformities. The concept of Developmental Origins of Health and Disease (DOHaD) supports that the initiation of major chronic diseases including diabetes begins in utero and in early development. Maternal hyperglycemia has been shown to affect fetal programming, and was linked to variable offspring diseases, such as obesity, diabetes, neurodegenerative and psychiatric diseases, and other^49–53^. Future work should therefore address the mechanisms by which a diabetic environment in utero alters the trajectory of offspring development. We also provide some preliminary evidence suggesting that removing conception and early preimplantation development from under the influence of a diabetic condition of the mother may be beneficial for embryonic/fetal development, a notion that requires future testing.

## Materials and methods

### Animals

Mice CD1 were used for all experiments. The mice were obtained at 3 weeks of age from the National Cancer Institute (Raleigh, NCI). The mice were fed *ad libidum* with a standard diet and maintained in a temperature- and light-controlled room (22 °C, 14L:10D) in accordance with the guidelines of the Laboratory Animal Services at the University of Hawaii and guidelines presented in National Research Council’s Guide for Care and Use of Laboratory Animals published in 1996 by Institute for Laboratory Animal Research (ILAR) of the National Academy of Science (Bethesda, MD). All experimental protocols are approved by the University of Hawaii Institutional Animal Care and Use Committee (protocol 06-010).

### Media

Mineral oil was purchased from FUJIFILM Irvine Scientific (Santa Ana, CA); pregnant mares’ serum gonadotrophin (eCG) and human chorionic gonadotrophin (hCG) from ProSpec (East Brunswick, NJ). All other chemicals were obtained from Sigma Chemical Co. (St Louis, MO) unless otherwise stated. Medium T6 was used for IVF, Hepes-buffered CZB (HEPES-CZB) for gamete handling^54,55^, and CZB medium^54^ for embryo culture. Both CZB and T6 were maintained in an atmosphere of 5% CO_2_ in air, and HEPES-CZB was maintained in air.

### Induction of diabetes

Diabetes were induced by a single intraperitoneal injection of streptozotocin (STZ) at a dose 200 mg/kg into 5 weeks old CD1 mice^56^. Five days before the STZ injection mice were weighted and divided randomly into study and control groups. On the injection day (Day 0), or in the evening of the preceding day of injection (Day − 1) the mice were weighted again and STZ dissolved in citrate buffer (pH 4.4) was intraperitoneally injected (study group); control mice were injected with an equal volume of buffer. In the days/weeks after injection mice were weighted and their submandibular blood glucose level was assessed using a glucometer (OneTouch Ultra 2 blood glucose meter). Starting at 21 days post STZ injection mice with blood glucose above $\ge$ 250 mg/dL were considered diabetic and were used as oocyte donors for in vitro fertilization or as surrogate mothers for embryo transfer. In most cases glucose level of mice used was above 400 mg/dL (Table S1 and S3).

### In vitro fertilization and embryo transfer

Sperm capacitation and IVF were performed as reported by us before^57^. Briefly, the oocytes were collected from females induced to superovulate with injections of 5 IU eCG and 5 IU hCG given 48 h apart. Epididymal sperm were collected by release from cauda epididymis directly into T6 medium and were capacitated for 1.5 h at 37 °C in a humidified atmosphere of 5% CO_2_. The gametes were co-incubated for 4 h. After gamete co-incubation, the oocytes were washed with HEPES-CZB, followed by at least one wash with CZB medium. Morphologically normal oocytes were selected for culture.

Fertilized oocytes (oocytes with two well developed pronuclei and extruded 2nd polar body) were cultured in 50 µL drops of CZB medium pre-equilibrated overnight with humidified 5%CO_2_ in air. The number of 2-cell embryos (fertilized) was recorded after 24 h in culture. The 2-cell embryos were either cultured in vitro to the blastocysts stage, with daily assessment of developmental progression, or were cryopreserved to be later used for embryo transfer. For embryo transfer, the 2-cell stage embryos were transferred to the oviducts (9 per oviduct) of CD-1 females mated during the previous night with vasectomized CD-1 males.

### Vitrification and thawing of embryos

Two-cell stage embryos were vitrified using a two-step exposure to equilibrium and vitrification solutions. The equilibrium solution (ES) consisted of Pb1 medium^58^ containing 0.3% bovine serum albumin (BSA), 7.5% dimethyl sulfoxide (DMSO), and 7.5% ethylene glycol (EG). The vitrification solution (VS) consisted of Pb1 with 0.3% BSA, 15% DMSO, 15% EG, and 0.5 M sucrose. Twenty embryos at a time were exposed to the ES for 2 min at 37 °C, then moved to VS and within 50 s placed onto fine plastic strip of Cryotop (Kitazato, Japan) and immersed directly into liquid nitrogen (LN_2_). Thawing of embryo was accomplished by transferring the plastic strip of cryotop as rapidly as possible directly from LN_2_ into 200 µl drop of 1 M sucrose solution at 37 °C, allowing the oocytes to sink out of the cryotop. Two minutes later, the embryos were transferred into 0.5 M sucrose solution for 3 min, then HEPES-CZB medium for 2 min, and finally into CZB medium. The embryos were kept in culture at 37 °C, 5% CO_2_ in air until transferred into a recipient mouse.

### Fetal assessment and tissue collection

Caesarean section was performed on day 18.5 of pregnancy. Fetuses and their placentas were weighted. The placentas were either stored at − 80 °C or fixed in 4% paraformaldehyde (PFA) for histological assessment. Each fetus was inspected for gross congenital defects based on the morphological assessment of diabetic embryos established by Wentzel et al.^59^, weighed, measured from crown to rump, and detached from its respective placenta. The placentas were weighted and measured (width and length, with a diameter established by averaging these two measures). Each fetus was then individually dissected, and tissue samples were collected for future analyzes. Tissues were washed in Dulbecco’s PBS (D-PBS), drained, and placed singly into tubes. Tail samples were collected for sexing and stored at − 20 °C. Whole blood samples were collected after decapitation into 0.5 mL tubes with heparin (1 µl of 1,000 U/mL heparin lithium salt in water) and stored at − 80 °C. Whole brain samples were stored at − 80 °C. Liver samples were divided, and half stored at − 80 °C and half fixed in 4% PFA. Heart samples were all fixed in 4% PFA.

### Genotyping

Fetuses were sexed by PCR to amplify a Y chromosome marker *Zfy*^60^, with myogenin (*Myog*) served as an amplification control^61^. Genomic DNA was obtained from each fetus and isolated using Qiagen DNeasy Tissue Kit (Qiagen, Valencia, CA). Polymerase chain reaction (PCR) analysis was performed using the following primers: Zfy-F: AAGATAAGCTTACATAATCACATGGA, ZFY-R: CCTATGAAATCCTTTGCTGCACATGT, Myog-F: TTACGTCCATCGTGGACAGCAT, Myog-R: TGGGCTGGGTGTTAGTCTTAT. The PCR conditions were as follows: initial denaturation at 94 °C for 3 min; followed by 30 cycles of denaturation at 96 °C for 10 s, annealing at 60 °C for 30 s and extension at 72 °C for 45 s, and a final elongation at 72 °C for 5 min. The amplification products were analyzed on ethidium bromide-stained 1.5% agarose gels.

### Experimental design and statistics

The experiments were designed to test for the effects of DM on fertilization and pre- and post-implantation embryonic and fetal development. To assess fertilization and pre-implantation diabetic (DM_O_) or non-diabetic (CON_O_) females were used as oocyte donors for IVF and embryos were cultured to blastocyst stage in vitro. To assess post-implantation development a reciprocal embryo transfer model was used, in which embryos produced with oocytes from DM_O_ and CON_O_ females were transferred to diabetic (DM_S_) or non-diabetic (CON_S_) surrogate mothers (Fig. 2). The following outcomes were measured: (1) Fertilization Rate: proportion of 2-cell embryos obtained from oocytes inseminated; (2) Developmental Rate In Vitro: proportion of blastocysts obtained from 2-cell embryos cultured; and (3) Developmental Rate In Vivo: proportion of fetuses from embryos transferred. The additional measures included female blood glucose level, female responsiveness to hormonal ovarian stimulation, oocyte number per female, fetal sex, weight and crown to rump length (CRL), placental weight and diameter, and incidence of fetal congenital abnormalities.

Two-way ANOVA with Bonferroni post-hoc test was used to compare body weight and glucose level increase overtime in STZ-injected and vehicle-injected females. Student's t-test was used to assess the differences between DM_O_ and CON_O_ oocyte donors in regard to in vitro fertilization and embryo development in vitro parameters. One-way ANOVA with Bonferroni post-hoc test was used to compare four experimental groups subjected to measures of developmental rate in vivo and assessment of proportions of fetuses and abortions, and fetal and placental measures. Sex ratio was assessed with Fisher’s Exact test. For statistical analyses, percentages were always transformed to angles.

Lack of statistical significance was reported when a test gave P > 0.05. Presence of statistical significance was noted a test showed P < 0.05. The computations were done using Excel or GraphPad Prism.

## Supplementary information

Supplementary movie S1

Supplementary information
