# Supplementary material for: Pregnancy environment, and not preconception, leads to fetal growth restriction and congenital abnormalities associated with diabetes
Source: Sci Rep. 2020 Jul 23;10:12254. doi: 10.1038/s41598-020-69247-w (PMC7378839; doi:10.1038/s41598-020-69247-w)
Supplement: Supplementary file 2 — Supplementary information [file 41598_2020_69247_MOESM2_ESM.pdf]

Supplementary Information

Pregnancy environment, and not preconception, leads to fetal growth restriction and congenital abnormalities associated with diabetes

Pai-Jong Stacy Tsai[1,2], Yasuhiro Yamauchi[1], Jonathan M. Riel[2], Monika A. Ward[1,3]

[1] *Institute for Biogenesis Research, John A. Burns School of Medicine, University of Hawaii, 1960 East-West Rd, Honolulu, Hawaii, 96822*

[2] *Department of Obstetrics, Gynecology, and Women's Health, John A. Burns School of Medicine, University of Hawaii, Kapi'olani Medical Center for Women & Children, 1319 Punahou Street, Honolulu, Hawaii, 96826*

[3] Corresponding author: Monika A. Ward, Institute for Biogenesis Research, John A. Burns School of Medicine, University of Hawaii, 1960 East-West Road, 96822, Honolulu, HI. Tel. (808) 956-0779, E-mail: mward@hawaii.edu

This PDF file includes:

Figures S1 to S3
Tables S1 to 3
Legend for Movie S1

Other supplementary materials for this manuscript include the following:

Movie S1

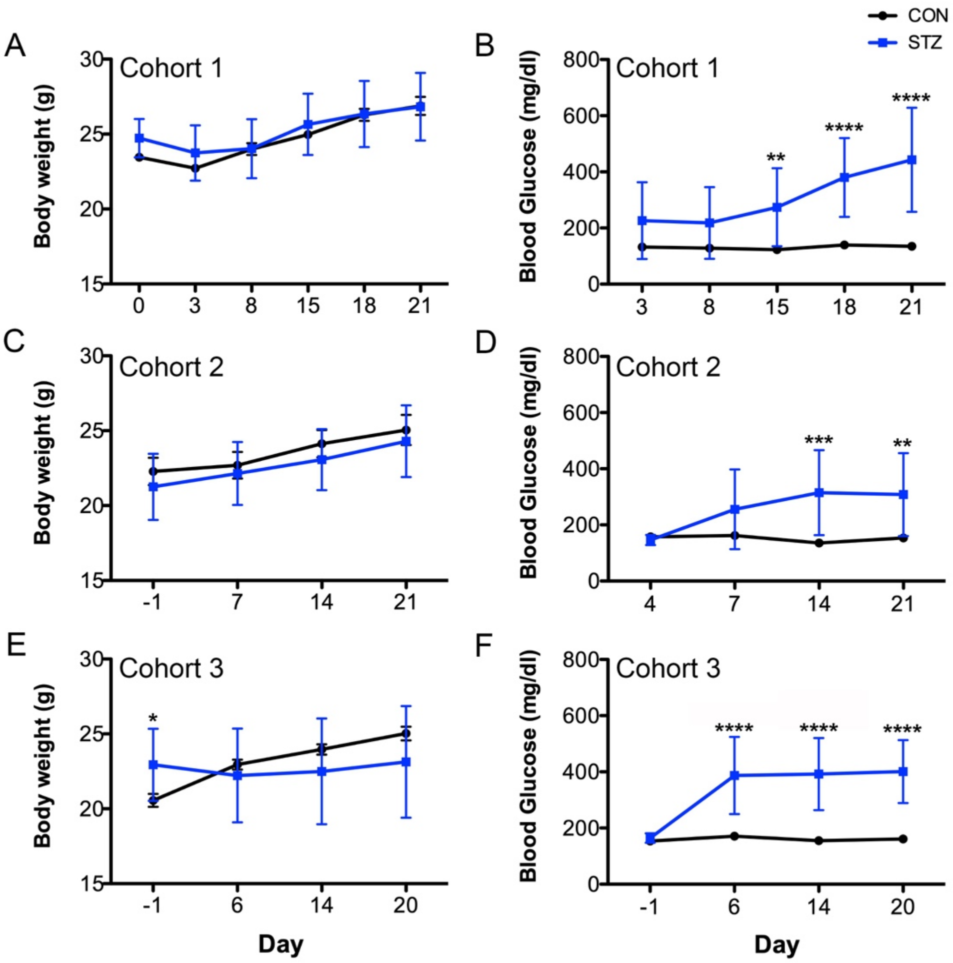

Figure S1. Body weight and blood glucose after STZ injection (Cohort 1). Mice were injected with STZ (STZ) or with vehicle (CON) on day 0 and their body weight (A,C,E) and blood glucose (B,D,F) were monitored over next 21 days. The data were analyzed with 2-way ANOVA with group (STZ and CON) and time (days -1 to 21) as factors, and post-hoc Bonferroni test for multiple paired comparison. In body weight analysis, in all 3 cohorts, both STZ and CON mice increased their body weight overtime (ANOVA, time: P<0.0001, P=0.0036 and P=0.0035, for Cohort 1, 2 and 3, respectively) but there were no differences between groups in ANOVA (group: P=0.094, P=0.1483, P=0.3474, for Cohort 1, 2 and 3, respectively). The interaction between factors was not affected in Cohort 1 and 2 (P=0.6840 and P=0.9877) but In Cohort 3 it was significant (P=0.0045) and the post-hoc test revealed a difference between groups one day prior to injection (*, P<0.05). In blood glucose analysis, significant differences between SZT and CON groups were observed in blood glucose level in all 3 cohorts: Cohort 1 (ANOVA, group: P<0.0001; time: P=0.0014; interaction: P=0.0042), Cohort 2 (ANOVA, group: P<0.0001; time: P=0.0304; interaction: P=0.0070), Cohort 3 (ANOVA, P<0.0001 for all) and the post-hoc test revealed that mice from STZ group had significantly higher levels of blood glucose starting from day 6 (Cohort 3) or 14-15 (Cohort 1-2) post injection and increasing thereafter (**, P<0.01; ****,****, P<0.0001). Graphs are average ± SDev with n=10 in each group in each timepoint for Cohort 1 and 2 and n=19-20 (STZ) and n=15 (CON) for Cohort 3.

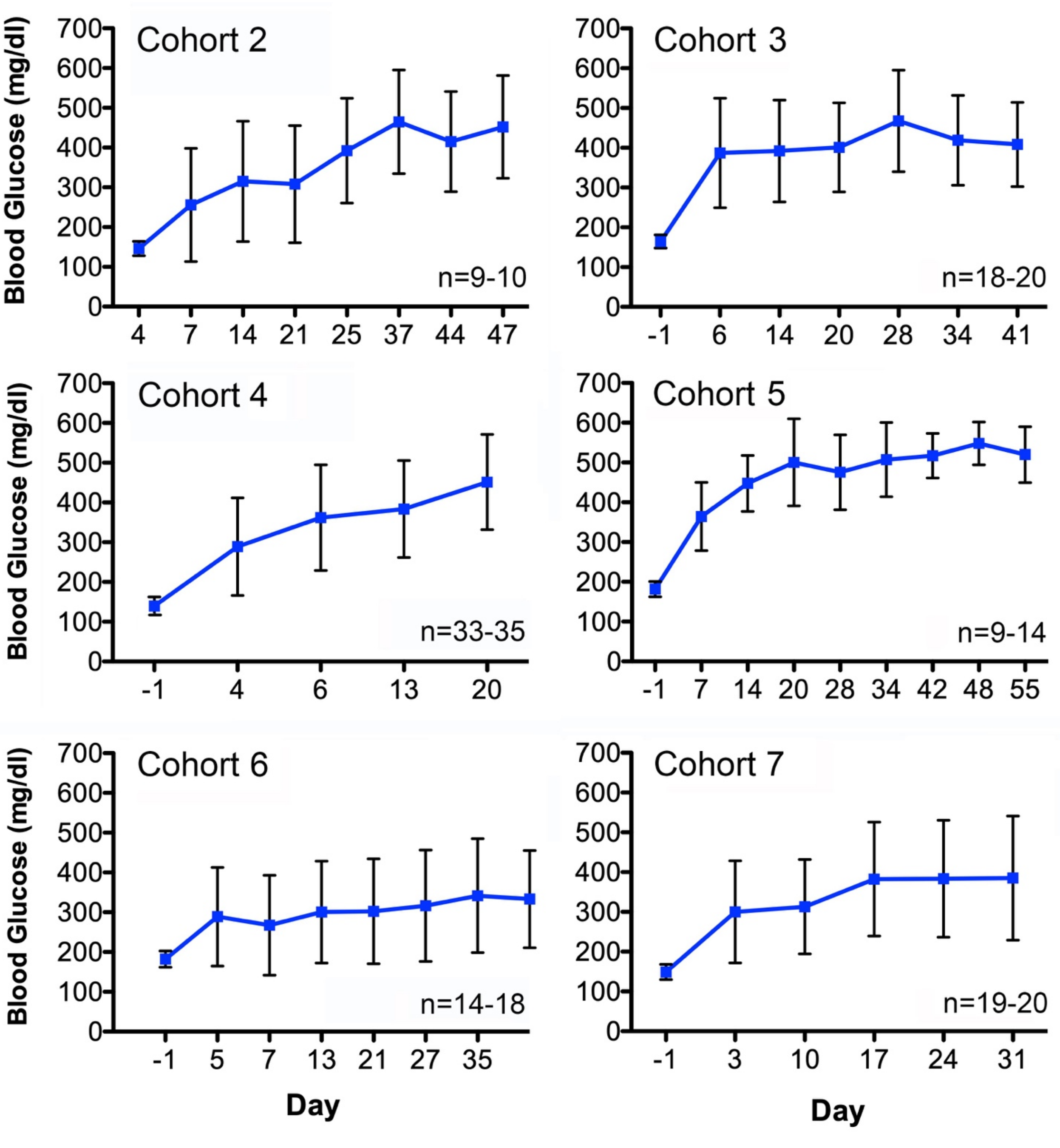

Figure S2. Blood glucose after STZ injection. Mice from 6 cohorts (Cohort 2 to 7) were injected with STZ (STZ) on day 0 and blood glucose was monitored over time. Single STZ injection resulted in increase of blood glucose that remained at high level for up to 55 days. Graphs are average ± SDev with n shown within each panel.

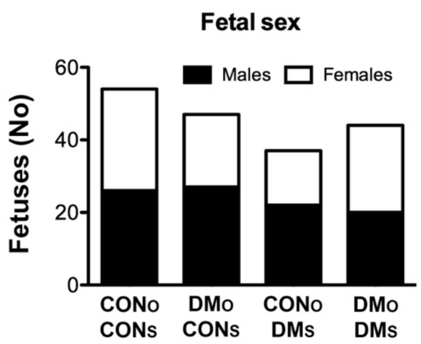

Figure S3. Sex distribution among fetuses. The sex distribution of male and female fetuses in each group. No statistically significant differences between groups were observed (Fisher's Exact test).

Table S1. In vitro fertilization and preimplantation embryo development in vitro.

Rep	Group	Female ID	Blood glucose (mg/dL)	No of oocytes	No of oocytes inseminated	2-cell embryos		Blastocysts	
						No	%[a]	No	%[b]
1	DM_O	1	496	7	5	0	0	0	0
		2	370	27	27	8	30	6	75
		3	452	16	16	5	31	4	80
		4	562	8	8	1	13	1	100
		5	317	26	26	19	73	18	94
	CON_O	1	132	2	2	0	0	0	0
		2	185	14	13	8	62	6	75
		3	135	10	8	4	50	2	50
		4	158	20	18	13	72	13	100
		5	150	23	22	17	77	9	53
2	DM_O	1	519	7	2	2	100	2	100
		2	486	18	18	17	94	17	100
		3	595	0	0	0	0	0	0
		4	471	23	23	22	96	22	100
		5	463	0	0	0	0	0	0
	CON_O	1	n/d	42	21	20	95	18	90
		2	n/d	46	42	40	95	37	93
		3	n/d	33	22	21	95	21	100
		4	n/d	28	27	27	100	26	96
		5	n/d	26	23	22	96	22	100
3	DM_O	1	491	0	0	0	0	0	0
		2	511	21	21	13	62	13	100
		3	505	0	0	0	0	0	0
		4	413	29	27	20	74	20	100
		5	427	0	0	0	0	0	0
		6	433	0	0	0	0	0	0
	CON_O	1	138	29	21	17	81	16	94
		2	143	17	13	11	85	11	100
		3	127	36	33	23	70	20	87
		4	119	16	14	11	79	11	100
		5	122	46	43	17	40	17	100

Diabetic (DM_O) and non-diabetic (CON_O) females were used as oocytes donors for in vitro fertilization. Percentage was calculated from [a] oocytes inseminated; [b] 2-cell embryos. The difference between the number of oocytes and the number of oocytes inseminated comes from exclusion of oocytes that were immature or deformed that was done after sperm were washed out at the conclusion of gamete-co-incubation. Females that did not yield any oocytes were assumed to be unresponsive to hormonal ovarian stimulation. The data summary and results of statistical analyses are shown in Figure 1.

Table S2. Effect of embryo cryopreservation.

Embryo type	No of 2-cell embryos transferred	Fetuses		Abortions		Fetuses with congenital anomaly	
		No	%[a]	No	%[a]	No	%[b]
Fresh	16	13	81	2	13	0	0
	16	15	94	1	6	0	0
	16	16	100	0	0	0	0
	48	***37***	***77***	***6***	***13***	***0***	***0***
Cryopreserved	16	13	81	2	13	0	0
	16	12	75	1	16	0	0
	16	12	75	3	19	0	0
	48	***44***	***92***	***3***	***6***	***0***	***0***

Percentage was calculated from: [a] embryos transferred; [b] fetuses. No statistically significant differences were noted between fresh and cryopreserved embryos that developed to live fetuses (T-test, P=0.115) or aborted (T-test, P=0.285).

Table S3. Post-implantation development.

Group	BG (mg/dL)	No of embryos transferred	Fetuses		Abortions		Fetuses with congenital anomaly	
			No	%[a]	No	%[a]	No	%[b]
CON_O-CON_S	96	18	11	61	1	6	0	0
	85	18	12	67	4	22	0	0
	121	18	7	39	8	44	0	0
	109	18	9	50	7	39	0	0
	147	18	15	83	2	11	0	0
DM_OCON_S	143	18	8	44	2	11	0	0
	125	18	11	61	6	33	0	0
	111	18	13	72	4	22	0	0
	82	18	15	83	3	17	0	0
	122	18	12	67	0	0	0	0
CON_ODM_S	600	18	14	78	1	6	1	7
	450	18	6	33	7	39	4	67
	588	18	8	44	6	33	1	13
	600	18	9	50	8	44	5	56
DM_ODM_S	496	18	11	61	5	28	1	9
	437	18	12	67	6	33	2	17
	559	18	9	50	8	44	1	11
	429	18	4	22	10	56	0	0
	600	18	8	44	7	39	3	38

BG, blood glucose prior c-section. Percentage was calculated from: [a] embryos transferred; [b] fetuses.

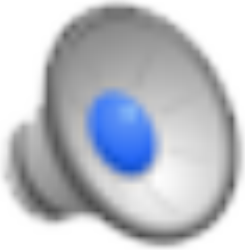

Movie S1. Heartbeat of fetus with multiple congenital defects.
